# *Sorbus gongshanensis* (Rosaceae), a new species from the Hengduan Mountains, China

**DOI:** 10.3897/phytokeys.144.48516

**Published:** 2020-03-17

**Authors:** Meng Li, Xin-Fen Gao, Jing Tian, Wen-Bin Ju

**Affiliations:** 1 Co-Innovation Center for Sustainable Forestry in Southern China, College of Biology and the Environment, Nanjing Forestry University, Nanjing, China Nanjing Forestry University Nanjing China; 2 Chengdu Institute of Biology, Chinese Academy of Sciences, Chengdu, China Chengdu Institute of Biology, Chinese Academy of Sciences Chengdu China; 3 Wuhan Botanical Garden, Chinese Academy of Sciences, Wuhan, China Wuhan Botanical Garden, Chinese Academy of Sciences Wuhan China; 4 Sino-Africa Joint Research Center, Chinese Academy of Sciences, Wuhan, China Sino-Africa Joint Research Center, Chinese Academy of Sciences Wuhan China

**Keywords:** Flora of China, taxonomy, Yunnan, Xizang

## Abstract

*Sorbus gongshanensis***sp. nov.**, a new species from the Hengduan Mountains China, is described and illustrated. It is similar to *S. kurzii* from China (Yunnan & Xizang), Nepal, and Sikkim in the size of the leaflets, glabrous veins, persistent (sometimes) herbaceous stipules and reddish brown villous inflorescences and red fruits, but differs in its serrate leaflet margins toothed in the distal half or often almost to their base, reddish brown villous to glabrous hypanthium and reddish brown villous infructescences, among other characteristics.

## Introduction

*Sorbus* L. *sensu lato* (*s.l.*; Rosaceae) comprises about 260 species distributed in the temperate zone of the Northern Hemisphere (Aldasoro et al. 1998; Lu and Spongberg 2003; Zika and Bailleul 2015; Sennikov and Kurtto 2017). Both molecular (Campbell et al. 2007; Lo and Donoghue 2012) and morphological evidence (Zheng and Zhang 2007) suggests that *Sorbuss.l.* is polyphyletic. *Sorbussensu stricto* (*s.s.*) includes ca. 80 species and is characterized by pinnately compound leaves. Recent molecular study of *Sorbuss.s.* suggests that the most recent common ancestor originated in eastern Asia (Li et al. 2017). The greatest diversity of species of *Sorbuss.s.* (ca. 60 species) is found in the mountains of southwestern China (principally the Hengduan mountains) and adjacent areas of Myanmar, Nepal, and the eastern Himalaya (Long 1987; Lu and Spongberg 2003; McAllister 2005; Watson and Manandhar 2012). This region is one of the world’s biodiversity hotspots, as it also is for the genus *Sorbus* (Myers et al. 2000; Li et al. 2017).

While studying *Sorbus* for the *Flora of Pan-Himalaya* Project, we found several accessions from Yunnan and Xizang, China, at the Harvard University Herbarium (GH) that were markedly different from other species of *Sorbus*. After detailed morphological examination, field investigation and literature study, it was concluded that these specimens represent an undescribed species, which we name *S. gongshanensis*. The description of *S. gongshanensis* is based on dried herbarium specimens stored at GH (all herbarium acronyms in this paper follow Thiers 2019).

## Material and methods

Morphological study was based on specimens deposited in the following herbaria: A, BM, CAS, CDBI, G, GH, K, KUN and NF. Macroscopic descriptions were based on the specimen sheets and notes made in the field. Detailed observations were conducted using an optical microscope. For scanning electron microscopy (SEM), dried pollen grains and stomata were mounted on metal stubs and sputtered with technical gold, and then were observed under Phenom proX SEM at 10 kV accelerating voltage at the Chengdu Institute of Biology, CAS. Pollen grains come from the field collection from Motuo [China, Xizang, *Meng Li 00281* (NF)]. Terminology of descriptive terms followed Flora of China vol. 9 (Lu and Spongberg 2003). Conservation assessment was based on the known distribution data and followed the IUCN red list category criteria (IUCN 2017).

## Results

### Taxonomic description

#### Sorbus gongshanensis

Taxon classificationPlantae

Xin-Fen Gao & Meng Li sp. nov.

2C5940AC-01DB-5AA8-B6D3-6ACB44F0ECA0

urn:lsid:ipni.org:names:77208266-1

Figs 1 , 2

##### Type.

China. Yunnan: Gongshan County, Bingzhongluo Xiang. Vicinity of Fucai, on the north side of Nianwaluo River, ca. 10.8 direct km of Bingzhongluo, east side of Gaoligong Mountains, 28°0.47'N, 98°31.11'E, alt. 2780 m, 1 Sept. 2006. *Gaoligong Shan Biodiversity Survey (2006) 31749* (holotype: GH; isotypes: CAS, KUN).

##### Diagnosis.

Similar to *S. kurzii*, but differs in its serrate leaflet margins toothed in the distal half or often almost to their base, reddish brown villous to glabrous hypanthium and reddish brown villous infructescences.

##### Description.

Shrubs or trees, 2–3 m tall. Bark gray. Branchlets tomentose when young, glabrous when old. Buds ovoid. Leaves pinnately compound, 8–10 × 5–5.5 cm; petiole 1.5–3 cm long; stipules membranous, caducous; rachis slightly winged, sulcate, sparsely tomentose; leaflets 2–4 pairs, opposite, elliptic, oblong to oblong-ovate, 2.8–3.5 × 1–1.5 cm, length/width ratio 2.4–3, surfaces essentially glabrous or sparsely (moderately) villous at flowering, usually glabrescent thereafter; blade paler abaxially, dull green adaxially; lateral veins 8–11 pairs, margins serrate, in the distal half or often almost to their base; base rounded or oblique, apex acute. Inflorescences corymbose, 4–5 × 2–3 cm, 3–15 flowered, sparsely reddish brown villous; stipules semi-orbicular, 0.5–0.8 × 1–1.3 cm, herbaceous, persistent in fruit; pedicels sparsely reddish brown villous. Flowers 6–8 mm in diam.; hypanthium reddish brown villous or glabrous, sepals 1–1.5(–2) mm long, margins entire; petals white, orbiculate to obovate, 3–4 mm long; stamens 15–20; carpels 1/2 adnate to hypanthium, styles 3–5. Infructescences sparsely reddish brown villous; pomes red, globose to subglobose, 6–8 mm in diam.; sepals inconspicuous, incurved when fruiting. Seeds brown, ovoid-lanceoloid, 3–4 × 1.2–1.5 mm, slightly asymmetric.

**Figure 1.**
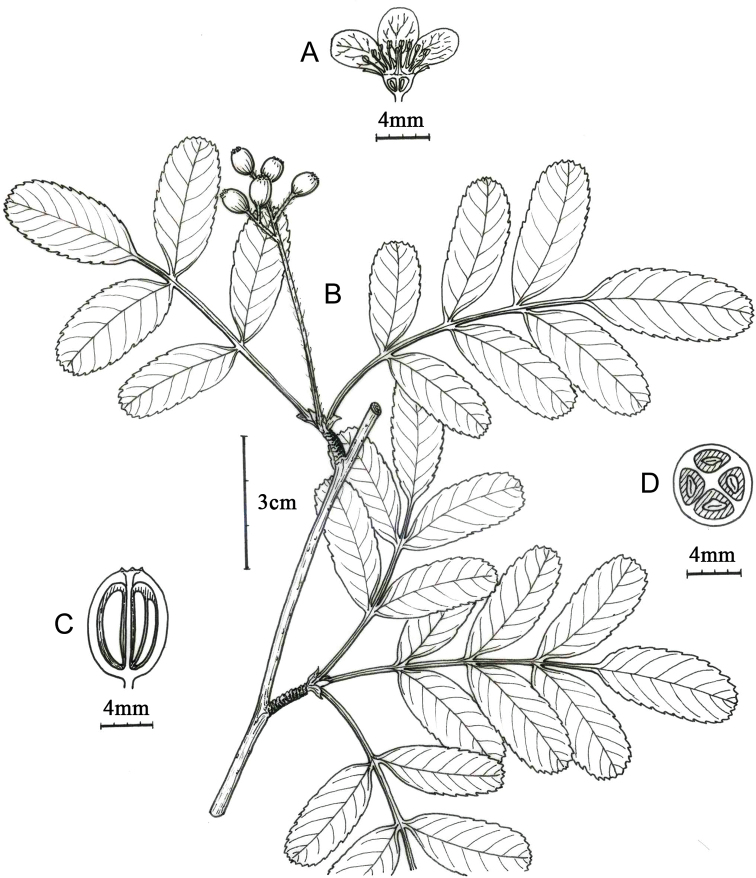
Main morphological characters of *Sorbus gongshanensis***A** flower, longitudinal section **B** fruiting branch **C** fruit, longitudinal section **D** fruit, cross section.

**Figure 2.**
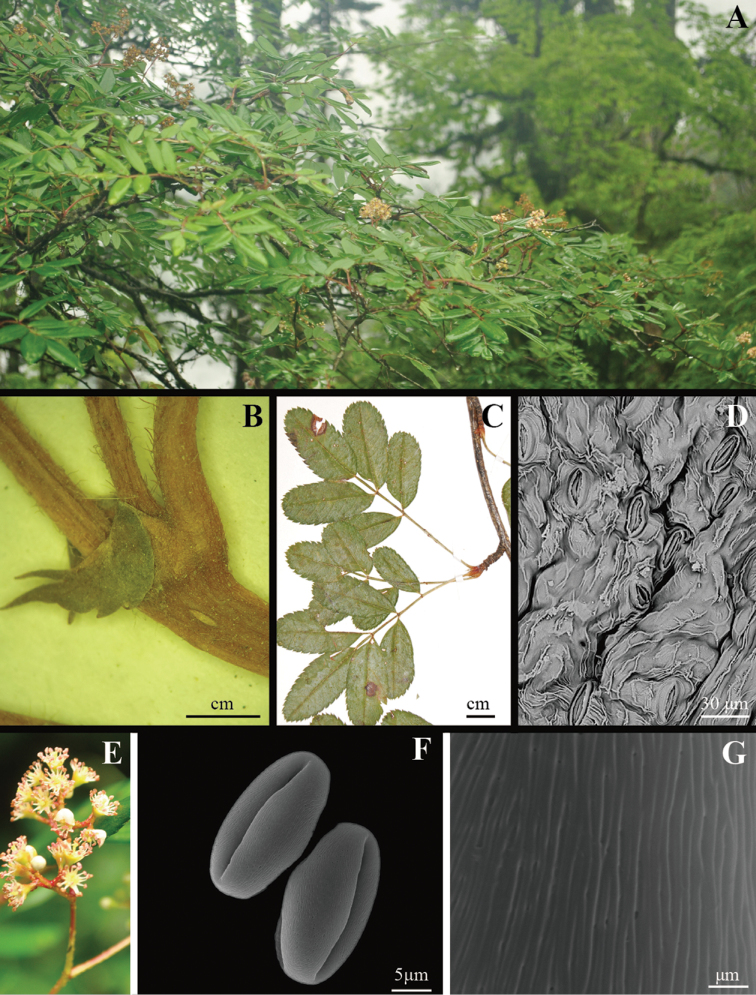
*Sorbus gongshanensis***A** habit **B** stipules and reddish-brown hairs on infructescences **C** leaves **D** stomata of abaxial blade surface **E** inflorescence **F** perprolate shape pollen, length of polar axis (33.03 ± 2.67 μm) and equatorial (16.61 ± 2.44 μm) diameter **G** striate-perforate ornamentation of pollen grains.

##### Etymology.

The specific epithet refers to the type locality, Gongshan County.

##### Phenology.

Flowering May–July, fruiting September–October.

##### Distribution and ecology.

**Figure 3.**
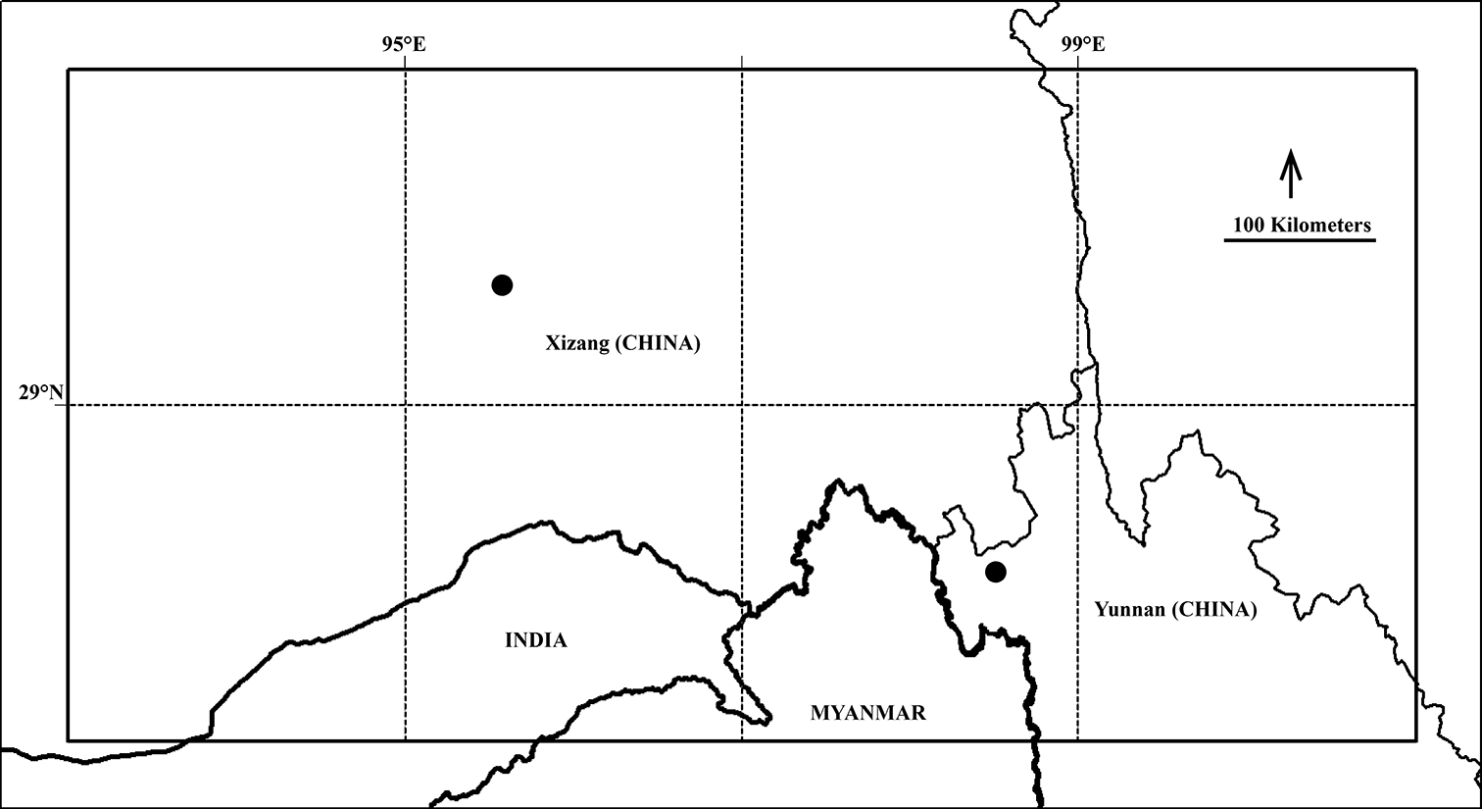
Geographical distribution map of *Sorbus gongshanensis* from Yunnan and Xizang province, China.

*Sorbus gongshanensis* is known from the Yunnan & Xizang Province, China (Fig. 3). It grows in broad-leaved forests or on rocky slopes; 2500–3000 m.

##### Specimens examined.

China. Yunnan: Gongshan County, Cikai Xiang, east side of Gaoligong Mountains, along the Danzhu River, on the roadside from Nu Jiang to Danzhu, 27°37.82'N, 98°37.30'E, alt. 2650 m., 2 July 2000. *Li Heng 11905* (GH, CAS, KUN). Xizang: Motuo County, 80k to Galung La, 29°42.30'N, 95°34.24'E, alt. 2782 m., 2 June 2015. *Meng Li 00281* (CDBI, NF).

##### Conservation status.

The distribution of *S. gongshanensis* is based on three collections. The collection notes mention that *S. gongshanensis* is occasional in forests among boulders. There is no direct or indirect information about its current conservation status or possible threats. We therefore assign the conservation status of *S. gongshanensis* as ‘Data Deficient (DD)’ according to the IUCN red list criteria (IUCN 2017).

## Discussion

*Sorbus* species show a high level of similarity in flower structure and color. The numbers of leaflets and fruit color are also fairly consistent across the group (Li et al. 2017). The number of leaflet pairs in *Sorbus* range between 2 and 21 pairs, and fruit color ranges between white, pink, red and orange-red (Lu and Spongberg 2003). While useful floral morphological characteristics are limited, pairs of leaflets, leaflet size, serra position, stipule shape and fruit color can provide valuable information for the identification of *Sorbus* at the species level.

**Figure 4.**
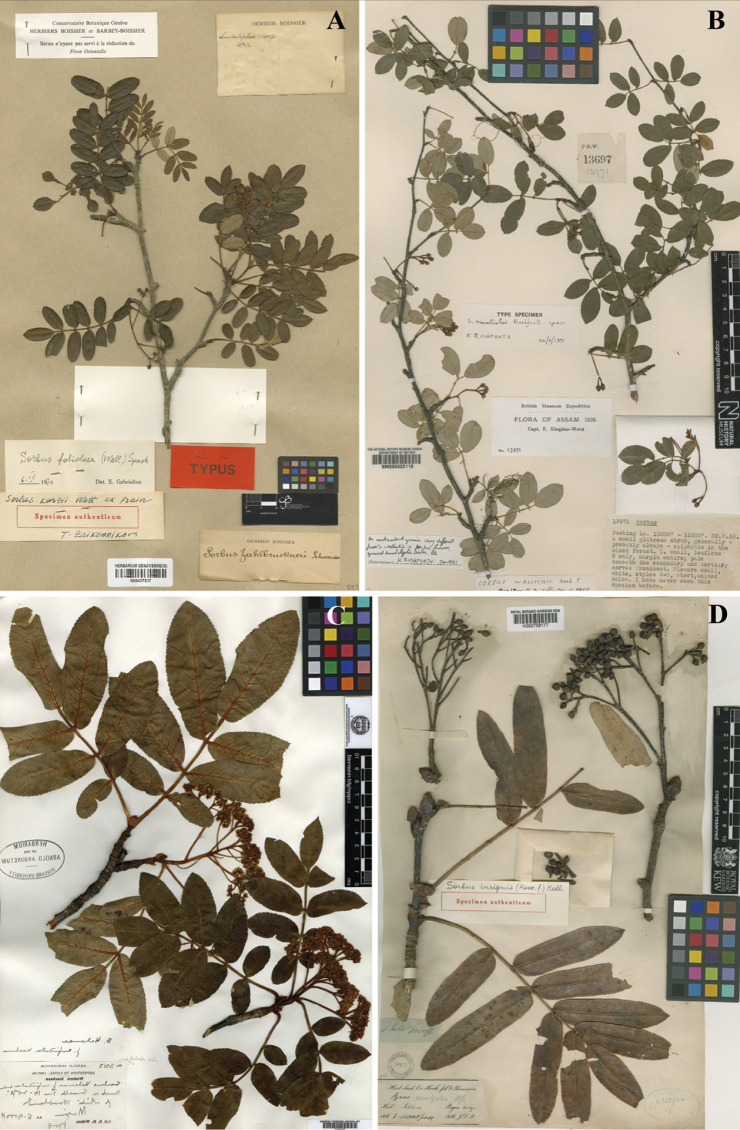
Type materials of four similar species distinguishing *Sorbus gongshanensis***A***Sorbus kurzii* (G barcode 00437217) **B***Sorbus macallisteri* (BM barcode 000602118) **C***Sorbus helenae* (A barcode 00046019) **D***Sorbus insignis* (K barcode 000758177).

A few species in the Hengduan Mountains have few pairs of leaflets. This group includes *S. helenae* Koehne (3–4 pairs), *S. insignis* (Hook. f.) Hedl. (3–6 pairs), *S. kurzii* (Watt ex Prain) C. K. Schneid (3–6 pairs) and *S. macallisteri* Rushforth (1–2 pairs) (Hedlund 1901; Hooker 1878; Koehne 1913; Prain 1904; Rushforth 1991; Schneider 1906) (Fig. 4 and Table 1). *Sorbus gongshanensis* is easily distinguished from all others by several distinctive characteristics (Long 1987; Lu and Spongberg 2003; Watson and Manandhar 2012) (Table 1). The red fruits distinguish *S. gongshanensis* from all species except *S. kurzii*. However, *S. gongshanensis* differs in its serrate leaflet margins toothed in the distal half or often almost to their base, the hypanthium reddish brown villous or glabrous and infructescences reddish brown villous. In all other species (exclude *S. gongshanensis* and *S. kurzii*), the fruits are typically white. *Sorbus gongshanensis* is also distinguished from *S. macallisteri*, *S. helenae*, and *S. insignis* by its persistent herbaceous stipules when fruiting (Table 1). Furthermore, *S. gongshanensis* have 2–4 pairs of leaflets which have serrate margins. *Sorbus macallisteri* only has 1–2 pairs of leaflets with few teeth. *Sorbus helenae* and *S. insignis* both have more pairs of leaflets (3–6 pairs) with the leaflets also longer and broader (10–20 cm long, 1.7–4 cm wide) than *S. gongshanensis* (Table 1).

**Table 1.** Comparison of characters distinguishing *Sorbus gongshanensis* and similar species.

	*Sorbus gongshanensis*	*S. kurzii*	*S. macallisteri*	*S. helenae*	*S. insignis*
Leaf & petiole length (cm)	8–10 & 1.5–2.5	7–11 & 1.2–2.8	3.5–6 & 1–1.8	13–20 & 2.5–4	10–15 & 1.7–3
Pairs of leaflets	2–4	3–6	1–2	3–4	3–6
Leaflet size (cm)	2.8–3.5 × 1–1.5	1.8–3.2 × 0.8–1.8	1.7–3 × 0.7–1.4	5–9 × 2–3	3–5 × 1–2
Margins	Deep toothed 1/2 way and often almost to base	Finely toothed in upper 1/2	Few teeth	Serrate or doubly serrate	Finely toothed in upper 1/2 or 1/4
Veins	Glabrous	Glabrous	Glabrous	Reddish brown villous along veins	Glabrous
Stipules	Herbaceous, semiorbicular, persistent	Herbaceous, lanceolate, persistent	Membranous, lanceolate, caducous	Membranous, lanceolate, caducous	Membranous, lanceolate, caducous
Inflorescences	Reddish brown villous	Reddish brown villous	Glabrous	Reddish brown villous	Slightly pubescent
Hypanthium	Reddish brown villous to glabrous	Glabrous to pubescent	Glabrous	Glabrous	Glabrous to pubescent
Infructescences	Reddish brown villous	Glabrous	Glabrous	Glabrous to pubescent	Glabrous
Fruits color	Red	Red	White	White	White

There are also several species sometimes with few pairs of leaflets found in other geographic regions. They are *S. gracilis* (Siebold et Zucc.) K. Koch [3–6 pairs of leaflets, distributed in Japan (Honshu, Shikoku, and Kyushu)], *S. sargentiana* Koehne (3–5 pairs of leaflets, distributed in southwest Sichuan Province, and northeast Yunnan Province), *S. sambucifolia* (Chamisso & Schlechtendal) M. Roemer (3–5 pairs of leaflets, distributed in Alaska, Japan, and the Russian Far East), and *S. sitchensis* M. Roem. (3–5 pairs of leaflets, distributed in the Pacific Northwest) (Chamisso and Schlechtendal 1827; Koch 1853; Koehne 1913; Roemer 1847; Siebold and Zuccarini 1846). *Sorbus sargentiana*, *S. sambucifolia*, and *S. sitchensis* have larger infructescences when fruiting (more than 30 fruits), while *S. gracilis* has large stipules (1.5 × 1.5 cm) at the nodes of the inflorescences. *Sorbus gongshanensis* can be easily distinguished from these four species by its few fruits and small stipule size.

## Supplementary Material

XML Treatment for Sorbus gongshanensis
